# PCBs and PCDD/Fs in Bluefin Tuna: Occurrence and Dietary Intake

**DOI:** 10.3390/ijerph15050911

**Published:** 2018-05-03

**Authors:** Grazia Barone, Arianna Storelli, Rita Garofalo, Rosanna Mallamaci, Nicoletta C. Quaglia, Maria Maddalena Storelli

**Affiliations:** 1 Biosciences, Biotechnologies and Biopharmaceutical Department, University of Bari “Aldo Moro”, Strada Prov. le per Casamassima Km 3, 70010 Valenzano (Ba), Italy; grazia.barone@uniba.it (G.B.); arianna.storelli@uniba.it (A.S.); rita.garofalo@uniba.it (R.G.); rosanna.mallamaci@uniba.it (R.M.); 2 Department of Emergency and Organ Transplantation, Section of Veterinary Clinic and Animal Production, University of Bari “Aldo Moro”, Strada Prov. le per Casamassima Km 3, 70010 Valenzano (Ba), Italy; nicolettacristiana.quaglia@uniba.it

**Keywords:** bluefin tuna, estimated dietary intake, PCBs, PCDD/Fs, risk assessment

## Abstract

Polychlorinated biphenyls (PCBs) and polychlorinated dibenzo-furans (PCDD/Fs) were measured in Mediterranean bluefin tuna (*Thunnus thynnus*) to verify the compliance with the EU regulations for food commercialization. The estimated intakes were also evaluated. The analyses were performed by gas chromatography-ion trap tandem mass spectrometry (GC-MS-MS). The PCBs were dominant (1132.0 ng g^−1^ l.w.), followed by PCDFs (23.2 pg g^−1^ l.w.) and PCDDs (8.5 pg g^−1^ l.w.). The pollutant levels (dl-PCBs: 0.7 pg TEQ/g w.w.; PCDD/Fs: 1.9 pg TEQ/g w.w.) and their sum expressed as TEQ values (2.6 pg TEQ/g w.w.) remained below the limits for human consumption proposed by the European Union. On the contrary, the sum of the six indicator non-dioxin-like PCBs (84.2 ng g^−1^ w.w.) was slightly above the maximum level fixed by the in-force legislation. The estimated dietary intakes for PCDD/Fs plus dl-PCBs were below the toxicological reference values (TRVs) set by various international bodies, while non-cancer and cancer risk assessment revealed a safety concern. Additionally, the estimated intake of ndl-PCBs exceeded the maximum levels set by different European countries. These findings suggest caution in tuna consumption together with an active and frequent surveillance of the chemical quality of its flesh.

## 1. Introduction

Polychlorinated dibenzo-*p*-dioxins (PCDDs) and polychlorinated dibenzo-furans (PCDFs), usually known with the term “dioxins” and polychlorinated biphenyls (PCBs) are a group of halogenated aromatic hydrocarbons exhibiting a high toxic potential. Their toxic action in humans, following chronic exposure, includes carcinogenic potency, immunotoxicity, and a range of negative endocrine effects related to reproduction [1]. The International Agency for Research on Cancer (IARC) classifies 2,3,7,8-TCDD, the most toxic of the dibenzo-*p*-dioxins together with 2,3,4,7,8-PeCDF and PCB 126 as group 1 carcinogens, meaning “known human carcinogens” [2]. More recently a whole PCB group was classified as carcinogenic for humans [3] too. Due to their characteristics of elevated chemical stability and persistence, these toxicants are present in all environmental media compromising absolutely the “health” of the environment and its biota. The Mediterranean Sea, almost entirely landlocked and with a limited water exchange, constitutes a site surely overexposed to anthropogenic pressure [4,5,6]. Bluefin tuna, *Thunnus thynnus* (Linnaeus 1758) is an important species within the Mediterranean ecosystems. Characterized by long lifespan and fast-growth, this top predator of the pelagic trophic web is susceptible to contain high concentrations of these lipophilic pollutants [6,7]. In addition, Bluefin tuna is a high-performance fish with very elevated metabolic and food intake rates and features enhancing contaminant bioaccumulation [8,9]. There is a well-established evidence that fishery products are one of the main sources of human exposure to these pollutants [10,11]. Therefore, the interest and concern about the chemical safety of fishery products has been and still remains a salient point for public opinion [12]. The worldwide food legislation has fixed limits on the concentration of these chemicals in seafood prior to marketing, as well as a relevant number of international regulatory bodies have set up fish consumption guidelines for consumer protection. Moreover, the World Health Organization (WHO) has recommended assessing human exposure to these lipophilic pollutants on a regular basis in order to evaluate either the human health risks or the time trends of exposure and the effectiveness of specific management measures. Consequently, the present study reports the results of congener-specific analysis of PCBs and PCDD/Fs performed on Mediterranean Bluefin-tuna specimens considered one of the most highly valuable fishery resources. In particular, it was verified whether the concentrations of these contaminants match the criteria set by the European Union (EU) regulation for commercialized food [13] or whether the estimated intake level satisfies the standards established by different food safety organizations [13,14,15,16].

## 2. Materials and Methods

### 2.1. Sample Collection

**Figure 1:**
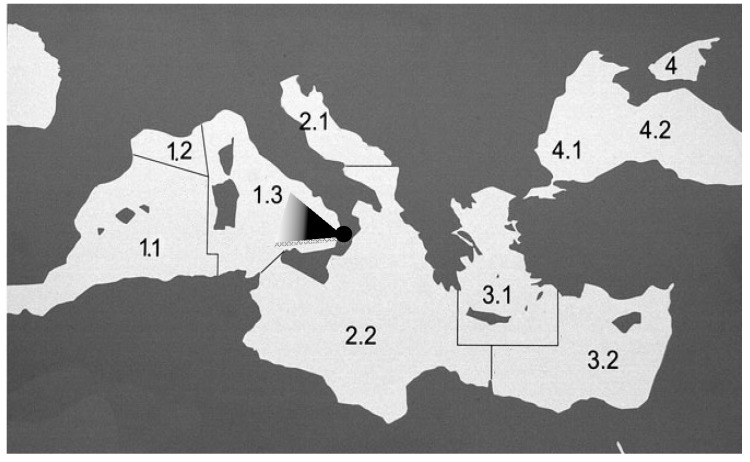
The sampling area: Food and Agriculture Organization (FAO) fishing area 37; division 37.1.3.

Twenty-six specimens of *Thunnus thynnus* (Bluefin tuna) (12 males and 14 females) were caught in the Southwestern Mediterranean Sea (FAO area 37.1) (Figure 1). Approximately 0.1–0.3 kg of muscle tissue was removed from the anterior portion of the carcass by a transverse dissection near the dorsal fin. These muscle portions were homogenized and kept in a deep freeze at −20 °C until chemical analysis. The sampling methodology used was in accordance with the European Commission Regulation [17]. The biological data of each fish were recorded (Table 1). The fish age and maturity stage were estimated according to Rodriguez-Roda [18] and Corriero et al. [19], respectively. The fish condition (K) was calculated using Fulton’s coefficient: K = W/L^3^ × 100 [20], where W is wet weight (g) and L total length (cm).

### 2.2. Chemical and Instrumental Analyses

All reagents and solvents were of analytical grade and were purchased from Merck (Darmstadt, Germany). The glassware was obtained from Levanchimica (Bari, Italy). PCB and PCDD/F standards were purchased from Wellington Laboratories (Guelph, Ontario, Canada). Eighteen individual PCB congeners (indicator non-dioxin-like PCBs (ndl-PCBs): 28, 52, 101, 138, 153, and 180 and dioxin-like PCBs (dl-PCBs): non-ortho PCBs 77, 81, 126, 169 and mono-ortho PCBs 105, 114, 118 123, 156, 157, 167, 189) and the seventeen 2,3,7,8-substituted PCDD/F congeners were quantified. A complete description of the experimental procedures relative to PCBs and PCDD/Fs has been described and validated elsewhere [21]. Approximately, 0.5 g of homogenized fish tissue was crushed in a mortar along with anhydrous sodium sulfate. The internal standard PCB 143 was added to the melted sample. The mixture extracted with hexane was concentrated under a gentle nitrogen stream. A gravimetric method was used to determine the percentage of fat in subsamples of the extract. At about 100 mg of the residual extract dissolved in hexane was applied to a silica clean-up column (8 g of acid silica in H_2_SO_4_, 44% wet weight 1/1, *v*/*v*) and eluted with 50 mL of a mixture of hexane/dichloromethane. After evaporation to dryness, 100 µL of iso-octane was added to the eluate and the sample was put into a GC-MS vial. The determination of PCDD/Fs was based on the US EPA method 1613. The samples were extracted with the method reported above and a multistep cleanup technique was undertaken to remove the matrix and the potential interfering components. The extract was first subjected to the action of the sulfuric acid to destroy the bulk of the fat and subsequently to a base back-extraction technique. The solution was then loaded onto a pre-conditioned florisil clean-up column and eluted with the appropriate solutions in order to remove the interfering components. The purified eluate containing PCDD/Fs was collected, concentrated, and fortified with appropriate C^13^-labeled extraction standards and transferred into a GC-MS vial. Finally, these obtained PCB and PCDD/F extracts were injected and analyzed separately on a Thermo Trace GC connected with a Thermo PolarisQ MS (Thermo Fisher Scientific, Waltham, MA, USA) operated in electron impact ionization (EI) mode. The chromatographic separation was attained by split-less injection on a capillary column with length of 30 m, i.d. 0.25 mm, and 0.25 µm thickness stationary phase film (RTX-5, Restek, US, Bellefonte, PA, USA) for PCBs, while for PCDD/Fs a capillary column with a length of 30 m × 0.25 mm and 0.25 µm thickness stationary phase film (RTX-200 Restek, US, Bellefonte, PA, USA) was employed. The MS was used in the SIM mode with the two most intensive ions of the molecular ion cluster monitored in specific windows for PCBs. PCDD/Fs quantification was archived using the ion trap in MS/MS mode and following the isotopic dilution method in accordance with US EPA 1613. The optimized parameters are shown in Table 2.

### 2.3. Quality Assurance and Quality Control

The quality control and quality assurance protocols included analysis of procedural blanks, duplicate samples, and the use of standard reference materials (CRM 349 for PCBs (cod liver oils) (BCR, Brussels) and CARP-2 for PCDD/Fs (NRCC) (IRMM, Geel, Belgium)) for each set of samples. For the replicate and standard reference materials, the relative standard deviations (RSD) were <10% for all the detected compounds. Moreover, the laboratory ability for measurements of the PCBs was validated in inter-laboratory trials organized by QUASIMEME (Laboratory Performance Studies). The results obtained were within a range of 20% from the consensus values. The multi-level calibration curves (*r*^2^ > 0.999) in the linear response interval of the detector were built in order to result in a range of 2–10,000 ng g^−1^ lipid weight for PCB 153, PCB 138 and PCB 180, in a range of 2–2000 ng g^−1^ lipid weight for the other PCB congeners and in a range of 0.02–2000 ng g^−1^ lipid weight for PCDD/Fs. The relative retention times (RRTs) of each analyte did not differ by more than ± 0.5% of the RRTs of the internal standards used for quantification, ion chromatograms and intensity ratios of the monitored ions. The relative intensities of the detected ions, expressed as a percentage of the intensity of the most abundant ion, were within 20% of the mean values obtained for calibration standards. All the procedural blanks were subjected to same processes of the samples and no measurable quantities of target compounds were detected. The limits of detection (LODs) were calculated as three times the signal-to-noise ratio and varied amongst analyte groups (00.1–0.26 pg g^−1^ for PCDD/Fs and 0.01–0.40 ng g^−1^ lipid weight for PCBs). Concentrations of PCBs and PCDD/Fs are presented as ng g^−1^ on a lipid weight basis and pg g^−1^ on a lipid weight basis, respectively. For comparative purposes, TEQ concentrations are expressed in pg TEQ g^−1^ wet weight.

### 2.4. Exposure Assessment

The estimated dietary intakes of PCDD/Fs plus dl-PCBs were calculated by multiplying the pelagic fish consumption data (85 g/week) [22] by the mean concentrations in tuna and then dividing by the body weight (70 kg) using the following equation:

Y = C × X/bw

where C is the sum of PCDD/F plus dl-PCB levels express as TEQ (pg TEQ g^−1^ wet weight), X is the consumption quantity of that particular item by an individual (g wet weight), and bw is the body weight of the individual. The TEQ concentrations were calculated by multiplying the individual congener concentrations by their respective toxic equivalency factors (TEFs), as established by the World Health Organization (WHO) in 2005 [23] and subsequently summed up to give the total concentrations. Non detected congeners were considered equal to zero (lower bound estimates). Risk-based consumption limits for non-cancer and cancer health endpoints, concerning PCBs and PCDD/Fs, were calculated using the EPA [24] developed fish consumption limit tables (Table 3). The variables used to calculate the fish consumption limits included the consumer body weight (70 kg), time-averaging period selected (monthly), fish meal size (227 g of cooked filet), number of monthly meals, and reference contaminant concentration ranges in the fish tissue calculated following the USEPA Integrated Risk Information System (IRIS).

### 2.5. Statistical Analysis

The Kruskal–Wallis test was conducted to verify the difference in the levels of PCB and PCDD/F accumulation, while the simple linear regression coefficient was used to examine the correlations between PCBs and specimen length. In order to investigate the influence of the size on PCB accumulation, the length of fish was chosen because it is less subject to fluctuation than body weight [25]. The level of significance was set at *p* < 0.05.

## 3. Results and Discussion

### 3.1. Concentrations and Congener Profiles of ndl-PCBs, dl-PCBs, and PCDD/Fs

The chemical analysis results of PCBs and PCDD/Fs together to detection frequency for each congener are illustrated in Table 4. As revealed by statistical analysis, PCBs were more abundant (range: 237.7–6673.5 ng g^−1^ lipid weight; mean: 1132.0 ng g^−1^ lipid weight) than the PCDD/F group (PCDD/Fs range: 5.3–70.1 pg g^−1^ lipid weight; mean: 31.7 pg g^−1^ lipid weight; PCDFs range: 2.7–67.5 pg g^−1^ lipid weight; mean 23.2 pg g^−1^ lipid weight; PCDDs range: ND-18.1 pg g^−1^ lipid weight; mean: 8.5 pg g^−1^ lipid weight) (*p* < 0.001) and no significant difference in the PCB and PCDD/F contamination level was observed between males and females (*p* > 0.05).

Regarding the detection frequency, congeners PCBs 138, 153, 180, and 118 occurred in all the examined samples, PCBs 52, 101, 114, and 123 were quantified in over than 80% of the samples, PCBs 77, 156, and 157 showed a detection frequency between 46.2% and 61.5%, while the rarest were PCBs 105 and 126 detected in 23.1% and 19.2% of the samples, respectively. The remaining PCBs (28, 81, 167, 169 and 189) were absent in all the samples tested. PCBs 153 (38.0%) and 138 (25.0%) were the most prominent congeners, followed by PCB 180 (14.5%), PCBs 101 and 118 which gave an almost equal contribution (8.0–8.1%), while congeners PCBs 52, 77, 105, 114, 123, 126, 156, and 157 collectively constituted a small fraction of the total (6.3%). A recent investigation on persistent organic pollutants in albacore tunas reported gender-related differences on the PCB profile [26]. A predominance of tri-to pentachlorinated congeners was observed in females, while males showed a higher contribution of hexa-to heptachlorinated PCBs [26]. In our case no variation in the isomeric classes of PCBs was observed, having both sexes a coincident fingerprint with a higher contribution of the middle chlorinated congeners (84.2%), followed by hepta-chlorine substituted PCB 180 (14.5%), while the low-chlorinated congeners made up a very small quota of the total PCBs (1.2%). This profile is coherent with that presented in various studies not only for tunas [7,10,26,27,28] but also for other fish [29,30] and marine mammals [31]. The predominance of the middle chlorinated congeners as well as the hepta-chlorine substituted PCB 180 occurs as a result of their stability because chlorine atoms in the 2,4,5-positions in one or both rings are particularly resistant to metabolic degradation by cytochrome P450 iso-enzymes [32], while the moderate presence of lower chlorinated PCBs has, generally, been explained by the elevated exchange rate of these congeners with the environment through the gills [27]. However, besides the physico-chemical properties of these compounds, a multitude of other ecological and biological factors, including the health conditions of animals, feeding habits, reproductive strategies, age, and size may control PCB accumulation. A rising bio-accumulation with the increasing of size has been noted many times in various marine organisms, including also tunas [5,8]. In this investigation no significant relationship was found between PCB concentrations and fish length, either considering sexes together (*R* = 0.17; *p* > 0.05) or separately (females: *R* = 0.10; *p* > 0.05; males: *R* = 0.26; *p* > 0.05). The absence of such a correlation may be explained by a growth dilution effect of contaminant load with increased size or age, reported above all in organisms such as tunas, which have a faster growth rate than the accumulation rate [33,34]. Additionally, reproduction may significantly influence the concentrations of these contaminants. At the beginning of the reproductive period, adult tunas use their stored lipids as an energy reservoir to produce gametes and consequently stored lipids in muscle decrease, leading to a loss of these lipophilic chemicals [26]. Different maturity stages of specimens here analyzed (see Table 1) might, thus, be the cause of the absence of PCB concentration/length relationships. In regards to the indicator ndl congeners, the relative concentrations ranged between 209.8 and 6042.2 ng g^−1^ lipid weight, with a mean value of 982.1 ng g^−1^ lipid weight, and their sum represented more than 85.0% of the total measured PCB concentration. In terms of the contribution to the total ndl-PCB levels, hexachlorobiphenyl PCB 153 was the most prevalent congener, accounting for 43.8% in good correspondence with what was reported in other surveys, demonstrating that PCB 153 has an average contribution of roughly one third to the sum of the six indicators [35,36]. The next dominant congener was PCB 138 constituting 28.9%, followed by PCB 180 making 16.7% and by PCBs 101 and 52 representing 9.2% and 1.3%, of the total burden respectively. The prevalence of congeners 138 and 153 is consistent with their chemical features as these molecules are less hydrophobic and not so tightly bound to sediment than higher chlorinated octa-deca-PCBs, the reason why they are more readily available to water organisms [37]. Concerning the 12 dl-PCBs, the levels were within the lipid weight range of 22.7–631.3 ng g^−1^ (mean: 149.9 ng g^−1^ lipid weight) and represented 13.2% of the total PCBs. Mono-*ortho* PCBs constituted more than 99.0% of dl-PCB levels. Of these PCBs, the congeners 118 (61.5%) and 156 (22.2%) were analytically predominant, followed by PCB 123 (8.3%), PCB 114 (3.3%), PCB 157 (2.7%), and PCB 105 (2.0%), which accounted for moderate percentages, while the coplanar non-*ortho* chlorine substituted PCBs 77 and 126 gave a negligible contribution (0.01%) to the total concentration of dl-PCBs. These findings are in accordance with other studies which report as predominant homolog group the pentachlorobiphenyls among dl-PCB, and in particular of PCB 118 not only in tunas [10,26,28,38] but also in other fish [30,38]. With respect to PCDD/Fs, the contribution of furans (70.9%) was higher than that of dioxins (29.1%), constituting more than twice in the total concentrations of PCDD/Fs (*p* < 0.01). The congeners that were detected more were 2,3,4,7-TCDD, 2,3,7,8-TCDF, 1,2,3,7,8-PeCDF, OCDD, and 2,3,4,7,8-PeCDF, having a detection frequency equal or above 50.0%. The other PCDD/F congeners quantified in more moderate amounts were 1,2,3,7,8-PeCDD (42.3%), 1,2,3,4,7,8-HxCDD (42.3%), 1,2,3,4,6,7,8-HpCDD (38.5%), 1,2,3,6,7,8-HxCDF (34.6%), 1,2,3,4,7,8-HxCDF (26.9%), and OCDF (23.1%), while the remaining ones were absent in all the samples examined. The profile of homologues was dominated by the tetra- to hexa-substituted PCDD/F congeners, whose combined levels accounted for more than 90.0% of all PCDD/Fs, while OCDD and OCDF constituted 3.6% and hepta-substituted PCDD/F congeners accounted for a small percentage of the total, representing 1.9%. It is not surprising that the lower substituted PCDD/F congeners dominated in the analyzed fish samples. In line with what was reported in the literature, the congener pattern changes in the food chain with the lower-chlorinated congeners dominating in species on the top trophic levels, including either marine mammals [39] or large predator fish such as tunas [40] and sharks [41]. This may be attributable to the limited biomagnification of higher chlorinated congeners along the food webs [42].

### 3.2. Compliance with European Union Legislation

Owing to the potential health risks of these substances, the maximum levels in fishery products have been established by various government agencies worldwide. European legislation has fixed the maximum levels of these contaminants, which are regularly updated according to the advances in scientific knowledge. The maximum levels for PCDD/Fs and PCDD/Fs plus dl-PCB compounds in seafood are set by European Union (EU) directive No 1259/2011 [13] and these levels cannot exceed a wet weight of 3.5 and 6.5 pg g^−1^ of toxic equivalents (WHO-TEQ), respectively. Moreover, the same regulation has set de novo a maximum level for the sum of the six ndl-PCBs in seafood, that is, a wet weight o f75 ng g^−1^, apart from some exceptions [13]. These different levels are necessary because the various PCB congeners exert their toxic effects via different mechanisms. In particular, PCBs may possess a dioxin-like PCBs or a non-dioxin-like PCBs activity. The 12 dl-PCB congeners share the same mechanism of action with the 17 toxic PCDD/Fs, whereby the effects are mediated through a specific cytoplasmic receptor protein, the aryl hydrocarbon (Ah) receptor [35]. The non-dioxin-like PCB group, including the six indicator congeners, appears to act via different modes exerting their toxicity primarily on neuronal cells with the reduction of dopamine neurotransmitter levels and interference with calcium homeostatis [43,44]. These congeners are less toxic than dl-PCBs, though they present a risk level not only due to their complex spectrum of adverse neurological effects, but also because of their presence as mixtures at higher concentrations in biotic and abiotic matrices [45,46]. In particular, the indicator congeners represent approximately half of the total non-dioxin-like PCBs existing in dietary products, and hence, have been recommended by the European Food Safety Authority (EFSA) Scientific Panel regarding Contaminants in the Food Chain (CONTAM Panel) as an appropriate marker of PCB contamination levels in food [35]. In the present study, the sum of the indicator ndl-PCB concentrations, given in wet weight (84.2 ng g^−1^ wet weight) to allow the comparison with the EU legislation, was slightly above the maximum level (75 ng g^−1^ wet weight) in many cases. In detail, 16 specimens displayed levels not compliant and of these, solely three presented concentrations close to the critical value. For the remaining 10 samples, the values were lower than the maximum level relative to marketing. On the contrary, the dl-PCB and PCDD/F level and their sum, expressed as TEQ values on a wet weight basis, remained lower than the maximum level fixed by the European Union’s (EU) regulations for food commercialization, being 0.7 pg TEQ g^−1^ wet weight, 1.9 pg TEQ g^−1^ wet weight and 2.6 pg TEQ g^−1^ wet weight, respectively.

### 3.3. Dietary Exposure Estimate

Fish consumption is a relevant source of polyunsatured fatty acids from the n-3 family, which are known to have a beneficial impact on human health by reducing the risk of coronary disease [47]. The American Heart Association (AHA) recommends consuming at least two 3 (2–85 g) oz portion of fish, especially fatty fish, weekly [48]. Nevertheless, the consumption of contaminated fish is one of the relevant pathways for the transfer of these contaminants from the environment to humans [49]. Consequently, the World Health Organization (WHO) sets toxicological reference values (TRVs) such as a tolerable daily intake (TDI) of 1–4 pg WHO-TEQ kg^−1^ body weight day^−1^ for PCDD/F plus dl-PCBs [14], while the European Scientific Committee on Food (SCF) defines a tolerable weekly intake level (TWI) to which humans can be exposed without harm to 14 pg WHO-TEQ kg^−1^ body weight [16]. Likewise, the Joint FAO/WHO Expert Committee on Food Additives [15] sets a provisional tolerable monthly intake (PTMI) of 70 pg WHO-TEQ kg^−1^ body weight month^−1^. In reference to these doses, the estimated mean intakes of PCDD/Fs plus dl-PCBs (EDI: 12.5% of TRV; EWI: 22.8% of TRV) were lower than the tolerable maximum levels. Additionally, the probabilistic P50, P75, and P95 intake estimates showed values of 2.6, 5.2, and 7.4 pg WHO/TEQ kg^−1^ bw per week, respectively. These values, being lower than 14 pg WHO/TEQ kg^−1^ bw computed by SCF, indicated that the dietary consumption of these marine products does not implicate an appreciable human health risk. Nevertheless, these results must be interpreted cautiously when we are thinking in terms of the risk for humans because the estimate intakes are point estimates, whereas the reference limits are defined for one’s life-long intake [50]. With regard to the non-cancer and cancer risks for the ingestion of PCBs and PCDD/Fs, the EPA guideline [24] presents data for the limits of fish consumption based on an exposure for a period of 70 years. Through the use of a so-called cancer slope factor (CSF) that considers the appearance of one new case of cancer for every 100,000 individuals significant, the EPA developed fish consumption limit tables that take into consideration both chronic and carcinogenic effects [24] (Table 3). On this basis, the monthly limit for tuna consumption was 1 portion of 227 g to avoid any carcinogenic risk for both contaminants, while no criticism was observed concerning to the noncancer risk for PCDD/Fs considering the value of reference (RfD: 0.7 pg/kg bw × day) derived by USEPA [51]. Similarly, no noncancer risk was evident for PCBs too, even though it is necessary to underline that the reference dose for chronic exposure set by EPA was fixed for Aroclor 1254 and not for PCB group analyzedhere, which might pose a larger risk. However, because it appears that it is safe to consume up to 8 meals/month the non-cancer risk was negligible. Concerning non-dioxin-like PCBs, the European Food Safety Authority (EFSA) has issued an opinion asserting that no health-based guidance value for humans can be established for these congeners because simultaneous exposure to non-dioxin-like PCBs and dioxin-like compounds hampers the interpretation of the results of the toxicological and epidemiological studies [35]. Consequently, uncertainty remains on how to interpret the data in terms of public health. However, the tolerable daily intake of 10 ng kg^−1^ bw day^−1^ formerly set at the national level in Netherland and then adopted also by the French Agency Food Safety (AFSSA) [52] and Norwegian Scientific Committee for Food Safety [53] can be of help as a guidance value. In our case, based on the mean ndl-PCB concentration of 84.2 ng g^−1^ wet weight, the dietary exposure was estimated at 14.6 ng kg^−1^ bw day^−1^, which was higher than the limit value reported above. This finding might be of particular concern considering that the recommended value set by Netherland refers not only to the indicator PCBs, but to the whole non-dioxin-like PCB congeners. In addition, humans exposed to a mixture of chemicals and the interaction with other toxicants may cause additive and synergic effects. It is the case of PCBs whose toxic action may be potentiated by mercury. In fact, it has been found that there is a positive relationship between Hg and PCB concentrations in fish, fish consumption by pregnant women, and deficit neurobehavioral development in children [54]. However, it is important to underline that different sources of uncertainties may affect the intake assessment. The expression of the results with non-detected equal to zero can cause an underestimation in the TEQ value and the FAO STAT database not reporting the data referring to tuna consumption but to the whole category of pelagic fish may lead to an overestimation of the exposure. Other uncertainties may be linked to food preparation, which may cause a under- or overestimation, due to lipid losses or further contamination in relation to the cooking mode (boiling, roasting, baking, grilling).

## 4. Conclusions

The scientific literature has indicated that the levels of these synthetic chemicals in the environment appear to have stalled in recent years [55,56], implying a consequent reduction in the dietary intake of PCBs and PCDD/Fs [57]. This finding surely reflects the entry in force of numerous legislative measures, for example, on emission of PCDDs, phasing out of PCBs, as well as the maximum tolerated levels in food, and so forth. In our study, the PCDD/F and PCB contents do not exceed the hygienic standards relative to marketing, while the ndl-PCB concentrations in many cases are higher than the maximum level established by EU [13]. The estimated dietary intakes of PCBs and PCDD/Fs are below the toxicological reference values set by the various international bodies. Nevertheless, the risk assessment presents a different scenario. The quantity of tuna safe to be consumed monthly, without causing chronic effects and carcinogenic risk because of the PCB content is moderate, while relative to PCDD/Fs, caution is recommended, especially for people from categories that are mostly exposed (fishermen and their families) or particularly sensitive (pregnant women, children, and elderly people). On the other hand, also for the six ndl-PCBs, the estimated intake is above the value set at the national level for non-dioxin-like PCBs in some countries, corroborating the opinion that hazards associated with consumption of this species cannot be ignored. As a final conclusion, although the decline in the human dietary exposure to these xenobiotic chemicals is a fact, high-trophic fish, especially from the Mediterranean Sea [6], continue to accumulate large amounts of chemicals from the environment polluted during the previous decades. Taking into account the relevant nutritional contribution that fish give to the diet and, on the other hand, that the consumption of contaminated fish is the major pathway of human exposure to these compounds, caution is suggested in tuna consumption and an active and frequent surveillance of the chemical quality of this valuable fishery resource is recommended.

## Figures and Tables

**Table 1:** The biological data of the Bluefin tunas examined.

Tunas No.	Sex	Body Weight (kg)	Body Length (cm)	Condition (K) ^1^	Estimated Age (years) ^2^	Maturity ^3^
1	F	18	107	1.47	2	Juvenile
2	M	30	115	1.97	4	Juvenile
3	M	28	120	1.62	3	Juvenile
4	M	35	120	2.03	3	Juvenile
5	F	38	123	2.04	4	Juvenile
6	M	40	127	1.95	4	Juvenile
7	F	43	129	2.00	4	Juvenile
8	F	45	130	2.05	4	Juvenile
9	F	43	131	1.91	4	Juvenile
10	F	51	133	2.17	4	Juvenile
11	F	50	135	2.03	4	Juvenile
12	F	50	135	2.03	4	Juvenile
13	F	50	135	2.03	4	Juvenile
14	M	56	140	2.04	5	Adult
15	M	56	140	2.04	5	Adult
16	M	56	140	2.04	5	Adult
17	F	64	143	2.19	5	Adult
18	F	51	143	1.74	5	Adult
19	F	54	144	1.81	5	Adult
20	F	50	147	1.57	5	Adult
21	F	69	150	2.04	5	Adult
22	M	69	150	2.04	5	Adult
23	M	76	155	2.04	5	Adult
24	M	93	172	1.83	6	Adult
25	M	102	178	1.81	7	Adult
26	M	161	218	1.55	10	Adult

^1^ [20]; ^2^ [18]; ^3^ [19].

**Table 2:** The optimized tandem mass spectrometry (MS/MS) parameters for the analysis of polychlorinated dibenzo-furans (PCDD/Fs) in gas chromatography mass spectrometry (GC/MS) PolarisQ used in MS/MS mode.

Compound		Precusor Ions (*m*/*z*)	Excitacion Voltage (V)	Product Ions (*m*/*z*)
TCDF	^12^C	306.0	4.75	241–243
	^13^C	318.0	4.75	252–254
TCDD	^12^C	322.0	4.4	257–259
	^13^C	334.0	4.4	268–270
PeCDFs	^12^C	340.0	5.05	275–285
	^13^C	352.0	5	286–288
PeCDD	^12^C	355.9	4.4	291–293
	^13^C	367.9	4.3	302–304
HxCDFs	^12^C	373.9	5.3	318–322
	^13^C	385.9	5.2	327–330
HxCDDs	^12^C	389.9	4.4	323–324
	^13^C	401.9	4.5	333–334
HpCDFs	^12^C	407.9	5.5	345–347
	^13^C	419.0	5.3	358–360
HpCDDs	^12^C	423.9	4.5	361–363
	^13^C	435.8	4.6	368–380
OCDF	^12^C	443.7	5.5	375–378
OCDD	^12^C	459.7	4.9	395–397
	^13^C	471.8	4.85	403–408

**Table 3:** The monthly fish consumption limits for carcinogenic and non-carcinogenic health endpoints ^1^.

Risk Based Consumption Limit 2	Polychlorinated Biphenyls (PCBs)		Dioxins/Furans (PCDD/Fs)	
	Non-Cancer Health Endpoints	Cancer Health Endpoints	Non-Cancer Health Endpoints	Cancer Health Endpoints
Fish Meals/Month	Fish Tissue Levels (μg g−1 w.w.)	Fish Tissue Levels (μg g−1 w.w.)	Fish Tissue Levels (μg g−1 w.w.)	Fish Tissue Levels (pg TEQ g−1 w.w.)
Unrestricted (>16)	0–0.0059	0–0.0015	NA	0–0.019
16	>0.0059–0.012	>0.0015–0.0029	NA	>0.019–0.038
12	>0.012–0.016	>0.0029–0.0039	NA	>0.038–0.05
8	>0.016–0.023	>0.0039–0.0059	NA	>0.05–0.075
4	>0.023–0.047	>0.0059–0.012	NA	>0.075–0.15
3	>0.047–0.063	>0.012–0.016	NA	>0.15–0.2
2	>0.063–0.094	>0.016–0.023	NA	>0.2–0.3
1	>0.094–0.19	>0.023–0.047	NA	>0.3–0.6
0.5	>0.19–0.38	>0.047–0.094	NA	>0.6–1.2
None (<0.5)	>0.38	>0.094	NA	>1.2

^1^ [24]; ^2^ the assumed meal size is 8 oz (227 g). None = no consumption recommended. NA = not available.

**Table 4:** The polychlorinated biphenyls (PCBs) (ng g^−1^ lipid weight) and the PCDD/Fs (pg g^−1^ lipid weight) concentrations and occurrence in Bluefin tuna from the Mediterranean Sea.

Congener	Min-Max	Mean ± St. Dev.	% Occurrence
% Lipid	10.2–40.2	19.0 ± 12.6	-
ndl-PCBs			
PCB 28	ND	ND	-
PCB 52	ND-87.8	13.2 ± 17.9	80.8
PCB 101	ND-590.6	90.6 ± 138.0	96.2
PCB 138	60.9–1730.1	283.5 ± 389.1	100.0
PCB 153	93.2–2501.6	430.5 ± 605.7	100.0
PCB 180	29.4–1132.0	164.3 ± 258.7	100.0
Σ ndl-PCBs	209.8–6042.2	982.1 ± 1041.0	-
Σ ndl-PCBs ^1^	3.4–219.3	84.2 ± 64.3	-
dl-PCBs			
Non-*ortho* PCBs			
PCB 77	ND-0.04	0.01 ± 0.01	46.2
PCB 81	ND	ND	-
PCB 126	ND-0.05	0.01 ± 0.02	19.2
PCB 169	ND	ND	-
Mono-*ortho* PCBs			
PCB 105	ND-25.7	3.0 ± 6.5	23.1
PCB 114	ND-9.4	5. 0 ± 3.1	80.8
PCB 118	22.7–528.9	92.2 ± 116.8	100.0
PCB 123	ND-30.8	12.4 ± 8.5	88.5
PCB 156	ND-100.0	33.3 ± 31.6	61.5
PCB 157	ND-9.5	4.0 ± 3.7	57.7
PCB 167	ND	ND	-
PCB 189	ND	ND	-
Σ dl-PCBs	22.7–631.3	149.9 ± 145.3	-
Σ dl-PCBs ^1^	0.9–46.6	17.4 ± 12.5	-
Σ ndl-PCBs + dl-PCBs	237.7–6673.5	1132.0 ± 1543.5	-
Σ ndl-PCBs + dl-PCBs ^1^	5.1–265.8	101.5 ± 76.9	-
Dioxins			
2,3,7,8-TCDD	ND-6.0	2.1 ± 1.7	80.8
1,2,3,7,8-PeCDD	ND-9.6	3.7 ± 4.6	42.3
1,2,3,4,7,8-HxCDD	ND-6.0	1.4 ± 2.3	42.3
1,2,3,6,7,8-HxCDD	ND	ND	-
1,2,3,7,8,9-HxCDD	ND	ND	-
1,2,3,4,6,7,8-HpCDD	ND-2.0	0.6 ± 0.9	38.5
OCDD	ND-1.6	0.7 ± 0.6	61.5
Σ PCDDs	1.0–18.1	8.5 ± 5.7	-
Σ PCDDs ^1^	0.02–4.1	1.5 ± 1.3	-
Furans			
2,3,7,8-TCDF	ND-27.0	9.2 ± 9.4	69.2
1,2,3,7,8-PeCDF	ND-6.9	2.4 ± 2.2	61.5
2,3,4,7,8-PeCDF	ND-25.3	8.2 ± 9.8	50.0
1,2,3,4,7,8-HxCDF	ND-4.8	0.7 ± 1.6	26.9
1,2,3,6,7,8-HxCDF	ND-10.4	2.2 ± 3.6	34.6
1,2,3,7,8,9-HxCDF	ND	ND	-
2,3,4,6,7,8-HxCDF	ND	ND	-
1,2,3,4,6,7,8-HpCDF	ND	ND	-
1,2,3,4,7,8,9-HpCDF	ND	ND	-
OCDF	ND-2.5	0.4 ± 0.9	23.1
Σ PCDFs	2.7–67.5	23.2 ± 20.3	-
Σ PCDFs ^1^	0.01–24.9	5.0 ± 6.8	-
Σ PCDD/Fs	5.3–70.1	31.7 ± 19.3	-
Σ PCDD/Fs ^1^	0.06–26.2	6.7 ± 7.0	-

^1^ ng g^−1^: wet weight; ND: not detected.
